# Prioritization of patients for germline testing based on tumor profiling of hematopoietic malignancies

**DOI:** 10.3389/fonc.2023.1084736

**Published:** 2023-01-30

**Authors:** Lucy A. Godley

**Affiliations:** Section of Hematology/Oncology, Departments of Medicine and Human Genetics, The University of Chicago, Chicago, IL, United States

**Keywords:** germline predisposition, tumor profiling, molecular profiling, hematopoietic malignancies, cancer risk

## Abstract

Germline predisposition to hematopoietic malignancies is more common than previously appreciated, with several clinical guidelines advocating for cancer risk testing in an expanding pool of patients. As molecular profiling of tumor cells becomes a standard practice for prognostication and defining options for targeted therapies, recognition that germline variants are present in all cells and can be identified by such testing becomes paramount. Although not to be substituted for proper germline cancer risk testing, tumor-based profiling can help prioritize DNA variants likely to be of germline origin, especially when they are present on sequential samples and persist into remission. Performing germline genetic testing as early during patient work-up as possible allows time to plan allogeneic stem cell transplantation using appropriate donors and optimize post-transplant prophylaxis. Health care providers need to be attentive to the differences between molecular profiling of tumor cells and germline genetic testing regarding ideal sample types, platform designs, capabilities, and limitations, to allow testing data to be interpreted as comprehensively as possible. The myriad of mutation types and growing number of genes involved in germline predisposition to hematopoietic malignancies makes reliance on detection of deleterious alleles using tumor-based testing alone very difficult and makes understanding how to ensure adequate testing of appropriate patients paramount.

## 1 Introduction

### 1.1 Opening case

**Figure 1:**
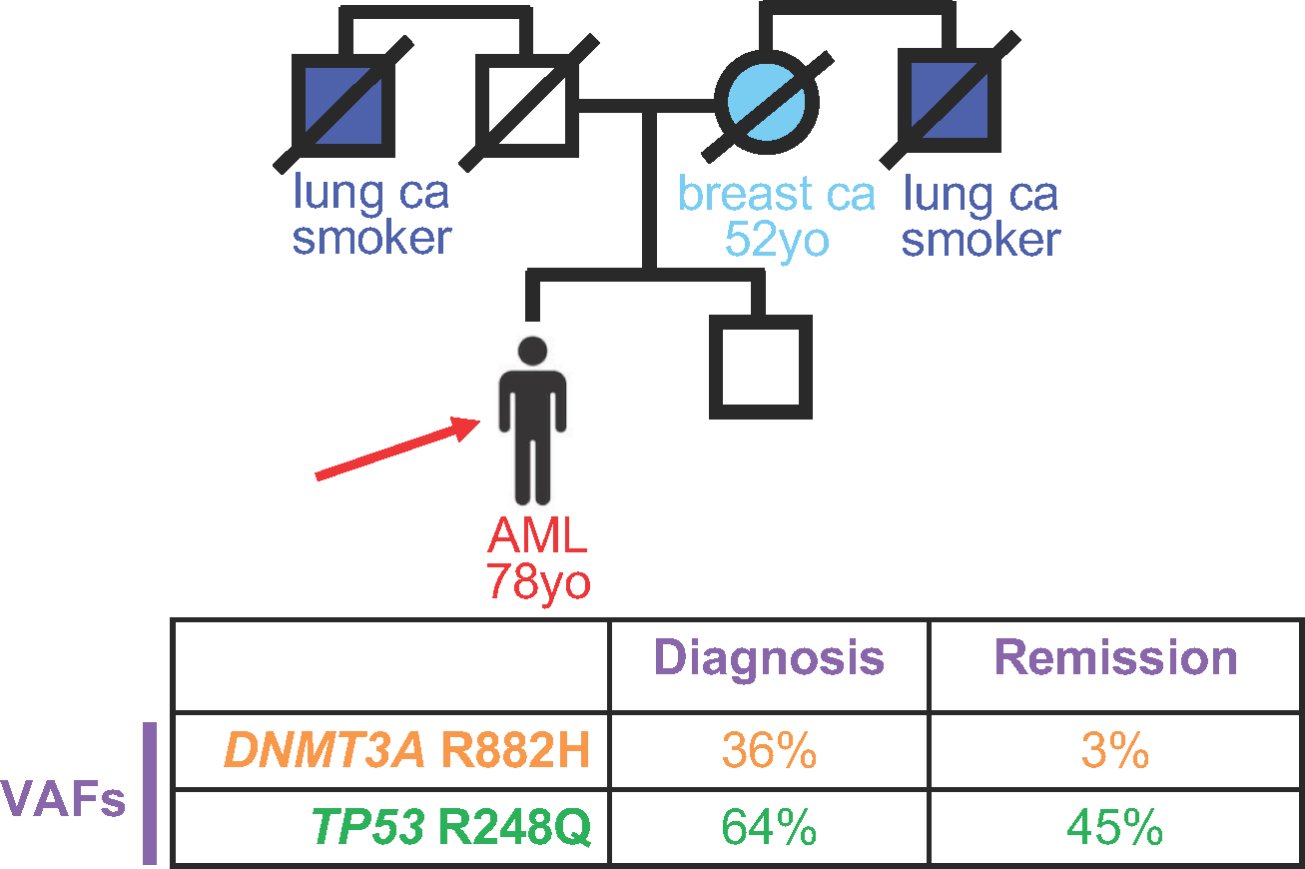
Persistence of deleterious variants in genes known to confer germline risk to hematopoietic malignancies suggests germline status. Top, Pedigree of an individual diagnosed with acute myeloid leukemia (AML) at 78 years old (yo), indicated by the red arrow. Circles, women; squares, men. Strike-out line indicates deceased individual at the time of pedigree generation. Ca, cancer. Bottom, Variant allele frequency (VAF) of deleterious variants at diagnosis (left) versus remission (right).

A 78 year-old (yo) man was diagnosed with acute myeloid leukemia during a work-up for his worsening fatigue (**Figure 1**). His family history was significant for his mother, who had been diagnosed with breast cancer at 52yo, and two uncles, who were smokers, with lung cancer. Cytogenetic analysis from the bone marrow at diagnosis revealed a normal karyotype, and molecular profiling demonstrated two DNA mutations: A *DNMT3A* mutation encoding R882H was present at a variant allele frequency (VAF) of 36%, and a *TP53* variant encoding R248Q was present at a VAF of 64%. The patient received standard induction chemotherapy with 7 days of cytarabine and 3 days of daunorubicin and achieved a clinical remission. Molecular testing from the remission bone marrow biopsy showed a decrease in the *DNMT3A* variant to 3%, but the *TP53* variant VAF remained high at 45%. Because the patient was in a clinical remission, with one DNA mutation decreasing to levels consistent with clonal hematopoiesis, the *TP53* variant VAF remaining about 50% suggested that this was a germline allele. The patient’s treating physician counseled the patient about this finding, and the patient chose to have a skin biopsy for germline genetic testing. The *TP53* variant was confirmed to be germline based on testing from DNA derived from cultured skin fibroblasts, and the patient was counseled about cancer risks associated with Li-Fraumeni Syndrome. Cascade testing for family members and cancer surveillance strategies were put into place for the patient and his affected family members.

## 2 Germline predisposition testing for patients with hematopoietic malignancies

Germline predisposition to hematopoietic malignancies (HMs) is being recognized increasingly (1, 2), as classification schemes (3, 4) and clinical guidelines (5–10) advocate for germline genetic testing for individuals with hematopoietic malignancies. Over time, such predisposition testing is being recommended for a larger and larger group of people. Currently, germline risk testing is advised for those with certain physical features (5, 11), and/or a: personal history of two or more cancers; personal history of a HM diagnosed at a much younger age than average [*e.g.*, MDS at <40yo]; personal history of a HM along with a family history of: another HM/prolonged cytopenia/or other hematologic abnormality (*e.g.*, macrocytosis) or diagnosis of a non-hematopoietic tumor in an individual < 50 yo within two generations of the proband; and/or molecular testing of tumor cells showing a deleterious variant in a gene known to confer a hereditary hematopoietic malignancy (HHM) at a VAF consistent with germline inheritance (1, 2, 5–8). VAFs in the range of 30-60% are generally considered typical for germline allele status, but this value can change depending on the testing platform and/or any copy number variants (CNVs) that may be present in the tissue being tested (7, 8, 12, 13). Other centers also prioritize those with excessive toxicity from chemo- radiotherapy for germline predisposition testing (11). Although germline predisposition testing is recommended for those diagnosed at particularly young ages as noted above, such testing should be considered in all patients with HMs regardless of age (14, 15). Certain cytogenetic and molecular abnormalities detected in tumor cells may also provide clues as to an underlying germline predisposition, including (i) the presence of two mutations within a gene known to confer inherited risk, such as *RUNX1* or *CEBPA*, one of which is actually a germline mutation; (ii) the presence of monosomy 7, which may suggest a deleterious germline variant in *SAMD9/SAMD9L* or *GATA2*; or (iii) a hypermutator tumor phenotype, which may indicate a germline alteration in a mismatch repair gene (16) or *MBD4* (17, 18).

Germline cancer syndromes were initially described by clinicians who naturally focused on extreme personal and family histories (5, 19). Thus, classic descriptions of these conditions were almost always too narrowly defined, as the Opening Case illustrates. The actual tumor spectrum of germline cancer disorders, like Li-Fraumeni syndrome, is likely much broader than first described (5, 19). As diagnosis becomes based more on molecular techniques rather than history and physical examination, we may identify more subtle cancer histories and/or physical findings associated with these classic cancer predisposition disorders.

## 3 Prioritizing DNA variants for germline testing identified in molecular profiling data from hematopoietic tumors

Because germline variants are present in all of the cells within a person’s body, malignant cells also contain that individual’s germline alleles (6–8, 12). Molecular profiling assays may be DNA- or RNA-based (13). In the case of platforms that use DNA derived from tumor biopsies, detected DNA variants may be derived from admixed normal cells and/or from germline alleles (12). Unfortunately, often, deleterious DNA variants are assumed to be somatic in nature and interpreted as such (20–22). Clinical reports may indicate that a particular variant could be germline in nature, but busy clinicians may not read these caveats closely or understand the distinction between germline and somatic alleles.

Importantly, the clinical classification of variants detected by tumor-based profiling are based on their assumed somatic nature, which may differ if the variant is actually germline (2, 8, 20–22). The impact of DNA changes is context dependent, and therefore, a germline allele, which is present in all tissues, may have different effects compared to a somatic allele, which is present only in a tumor (23, 24). For this reason, germline and somatic variant curations are distinct (23, 25). Recognizing this, it is our practice to review all DNA variants called in genes known to confer risk for HHMs from molecular testing of malignant hematopoietic tumor cells with the goal of identifying those that could be germline (6–8, 21). Particular gene alleles are overwhelmingly likely to be germline (**Table 1**), and identifying these in tumor cells quickly singles out these individuals for counselling regarding the likely germline nature of the allele (6, 21). When DNA variants likely to be germline are found in tumor-based sequencing assays, we notify the clinic physician in charge of the patient and urge genetic counselling and testing. Some centers have established parallel pipelines that assess for somatic and germline variants simultaneously by sequencing DNA from tumor cells and buccal swabs as well as tumor-derived RNA (13).

**Table 1:** Gene alleles that are commonly germline.

	Alleles that are overwhelmingly likely to be germline
***CHEK2*** NM 007194.4	• c.470T>C, p.1200T • c.1100delC, p.T367fs • c.1283C>T, p.S428F
***DDXI*** NM 016222.3	• truncating variants • c.3G>A, p.M1?

**Figure 2:**
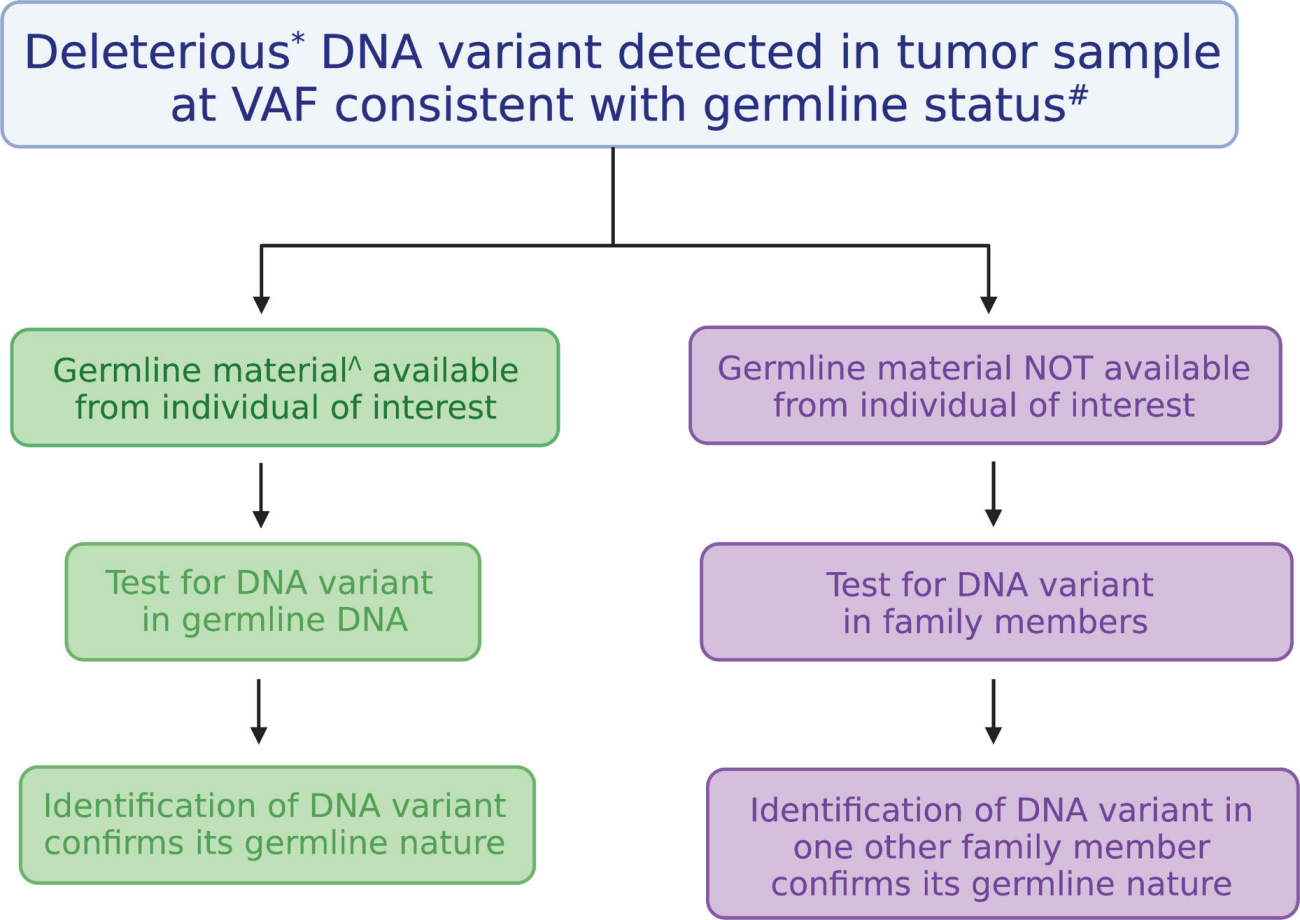
Algorithm for testing the germline nature of a DNA variant identified in a tumor. Testing the germline nature of a DNA variant begins with recognizing a deleterious DNA variant found in a gene known to confer cancer risk at a variant allele frequency (VAF) consistent with a germline allele. Testing can be performed using another DNA sample derived from a tissue considered equivalent to the germline (on left, in green) or through familial segregation (on right, in purple). Figure was generated with BioRender. *Deleterious variants are those classified as pathogenic or likely pathogenic. ^#^VAFs from 30-60% are generally considered to be consistent with germline status, but they can be as high as 100% depending on chromosome gains or losses. ^^^DNA derived from cultured skin fibroblasts, hair bulbs, or bone marrow-derived mesenchymal stromal cells are considered equivalent to germline samples.

As indicated in **Table 1**, based on our clinical experience at The University of Chicago, there are two genes in which certain variants are overwhelmingly likely to be germline: *CHEK2* and *DDX41*. When these variants are detected even in a single sample, they are typically seen at germline-range VAFs, and treating physicians are notified of the likely germline nature of the variants. We advocate genetic counselling, with recommendation for determination of germline status using either (i) testing of DNA from cultured skin fibroblasts or hair bulbs, both of which are equivalent to germline, or (ii) segregation of the variant within the family (**Figure 2**). Identifying a DNA variant in two related individuals is sufficient to determine germline status, and occasionally, this approach is more feasible than testing through skin biopsy and fibroblast culture. We advise against assuming that these variants are germline and do not provide clinical recommendations unless these variants have been confirmed to be germline. Importantly, founder mutations exist in other cancer predisposing genes, such as *BRCA1/2* and *TP53*, which may also confer risk to HMs as well as solid tumors. For this reason, care should be given to review all DNA variants in cancer predisposing genes when tumor based molecular profiling is performed.

Molecular profiling in patients with HMs is often conducted sequentially over time to document remission status. In these cases, serial sampling over time is an excellent means of prioritizing patients for germline testing (12), as outlined by the Opening Case. Gene variants that persist over time despite changes in disease status, especially those that remain in germline-range VAF from diagnosis through clinical remission, are likely to be germline, and again identify individuals who deserve genetic counseling and testing (12). However, we need to be cautious and interpret variants within the clinical context.

## 4 Case 2: After allogeneic hematopoietic stem cell transplantation (HSCT), molecular profiling can identify donor-derived germline variants

**Figure 3:**
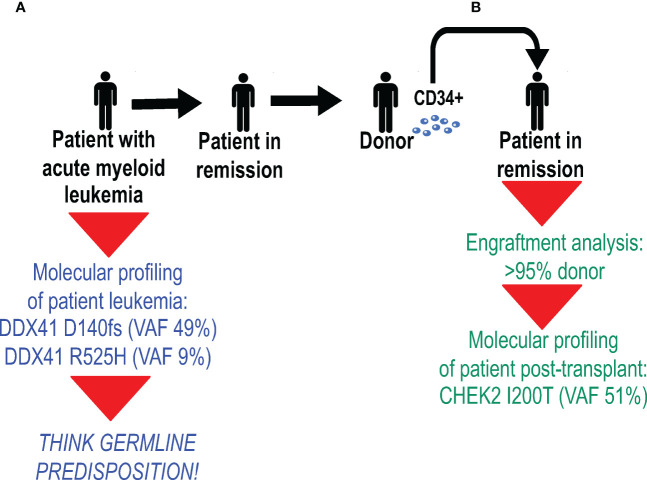
Molecular profiling has the capacity to identify donor-derived germline variants after allogeneic hematopoietic stem cell transplantation (HSCT). **(A)** Molecular profiling of a patient with acute myeloid leukemia revealed two deleterious DDX41 variants (shown in blue): the D140fs variant at a variant allele frequency (VAF) of 49%, and the R525H variant at a VAF of 9%. Because the D140fs variant has always been seen as a germline variant (**Table 2**), the patient had proper germline testing, which confirmed its germline status. **(B)** After allogeneic HSCT from an unrelated donor, a day 30 bone marrow biopsy was performed. Studies performed on bone marrow cells (shown in green) included engraftment analysis, which showed that >95% of bone marrow cells were donor-derived, and molecular profiling identified the CHEK2 I200T variant at a VAF of 51%.

The pre-transplant work-up of a patient with acute myeloid leukemia revealed two deleterious *DDX41* variants: one encoding the truncating D140fs variant with a VAF of 49%, and the other encoding the R525H variant at a VAF of 9% (**Figure 3**). Because the D140fs variant has always been seen as a germline variant (**Table 1**), the patient was counseled and proper germline testing confirmed its germline status. The transplant team decided to proceed with an allogeneic HSCT from an unrelated donor. The post-transplant day +30 bone marrow biopsy was performed, and several studies were performed in parallel: engraftment analysis [to determine the degree of donor chimerism], which showed that >95% of bone marrow cells were donor-derived; and molecular profiling [to ensure molecular remission from leukemia], which identified the CHEK2 I200T variant at a VAF of 51%, an allele which is overwhelmingly likely to be germline (**Table 1**).

## 5 Sensitivity around molecular testing after HSCT

In Case 2, the patient and his treating team did not have consent from the unrelated donor to know their germline genetic testing result, nor was there a mechanism to share this information with the donor. It is important to recognize that this situation is not unique to unrelated donors. Since all people have deleterious germline DNA variants, all donor types (*e.g.*, related, unrelated, umbilical cord) have the capacity to introduce such alleles into a transplant recipient. Sequential testing in which engraftment analysis is performed first and informs subsequent testing and reporting could have avoided this situation. If chimerism were tested first and the sample were noted to be overwhelmingly donor-derived, then reporting of molecular profiling could have noted that fact, at a minimum, or ideally, been restricted to leukemia-associated somatic variants. Limiting post-transplant clinical reports to detailing the presence or absence of malignancy-associated somatic DNA variants (*i.e.*, in this case, the DDX41 R525H allele) is appropriate, but requires coordination across clinical laboratories. Ideally, test reports should also clearly indicate that by the nature of this type of testing, germline variants of donor or host origin could be missed. Providers and patients receiving these reports need to know this information, as it could be clinically relevant over time for the patient or a related donor.

## 6 Cautions in using tumor profiling data

Although tumor-based molecular studies can identify some deleterious germline variants, these platforms should not be used in place of proper germline testing (**Table 2**). First, the ideal sample type for each test is distinct. For tumor-based testing, a sample containing tumor cells must be used. Therefore, peripheral blood with circulating malignant cells; involved bone marrow, lymph node(s), and/or cerebrospinal fluid; or any other tissue (*e.g.*, extramedullary hematopoiesis, myeloid sarcoma) containing such cells can be used to generate DNA. In most of these cases, normal cells are also present, with the quantity dictated by the degree of tumor burden. In contrast, germline testing is performed ideally using tissues that are equivalent to germline. Most clinical laboratories accept DNA derived from cultured skin fibroblasts, and some accept DNA generated from non-hematopoietic hair bulbs or bone marrow-derived mesenchymal stromal cells (MSCs), which are easily cultured from a bone marrow aspirate.

**Table 2:** Contrasting tumor-based versus germline testing.

	Tumor-based Testing	Germline Testing
Sample type	• peripheral blood • bone marrow • lymph node • CSF • any sample with hematopoietic tumor cells	• cultured skin fibroblasts • hair bulbs • bone marrow-derived mesenchymal stromal cells
Benefits	Sample is likely already being collected for other tests.	Result is confirmed germline and can be interpreted as such. Result is immediately relevant when considering relatives as allogeneic hematopoietic stem cell donors.
Cautions/ caveats	Hematopoietic tissues undergo somatic reversion easily, so the absence of a finding does not give assurance that there is no deleterious germline variant Non-coding regions of genes are typically not covered by these assays. CNVs are typically not covered by these assays.	Time from sample collection to result can take up to three months, which can complicate planning future therapy, including allogeneic stem cell transplantation.
Platforms	Generally cover genes/exons where deleterious germline variants can be found. Are typically designed to detect SNVs and are capable of detecting large CNVs, but are insensitive to small CNVs. Coverage depth is in the hundreds-thousands depth to allow detection of small clone sizes.	Generally are designed to cover genes/exons as well as non-coding regions (*e.g.*, prornoters and enhancers) where deleterious germline variants can be found. Are capable of identifying SNVs and CNVs. Current platforms need to be flexible to accommodate the predisposition genes that continue to be discovered. Coverage depth 30-50-fold is sufficient to detect germline-range VAFs.
Specific alleles	Often, the same allele (*e.g.*, in *TP53*, *RUNX1*, and *CEBPA*, among others) can be somatic or germline.	Specific alleles (*e.g.*, in *CHEK2* and *DDX41*) are overwhelmingly likely to be germline.

CNV, copy number variant; CSF, cerebrospinal fluid; indel, insertion/deletion; SNV, single nucleotide variant; VAF, variant allele frequency.

The distinction between the use of hematopoietic versus non-hematopoietic tissue for proper germline predisposition testing is of paramount importance. Use of non-hematopoietic tissue is critical for germline risk assessment, because hematopoietic tissue undergoes somatic reversion relatively easily compared to other tissues, like skin fibroblasts (19). For some deleterious germline variants, like those in *SAMD9* and *SAMD9L*, somatic reversion is a common mode of escape hematopoiesis (19). In these cases, correction in hematopoietic tissues occurs commonly, and therefore testing for these alleles in hematopoietic tissues fails to reveal the underlying germline defect. Somatic reversion has been documented for nearly all of the genes that confer germline susceptibility to HHMs. Thus, if hematopoietic tissue is used for germline genetic testing and a negative result is obtained, this may be a false negative result, and one cannot be confident that a deleterious variant is not present (19).

Traditionally, germline genetic testing has used peripheral blood or saliva/buccal swab for testing. However, we now recognize how frequent clonal hematopoiesis and therefore somatic mutation occurs within the hematopoietic compartment, which complicates DNA variant interpretation. For example, there have now been many cases of “mosaic *TP53* mutations” being confirmed to be due to CH (26). CH is itself a form of somatic mosaicism but one that arises well after embryogenesis and generally during adulthood. Therefore, we avoid germline genetic testing from hematopoietic tissues.

Thus, for these reasons, we recommend germline testing using DNA derived from tissues considered equivalent to germline (*e.g.*, cultured skin fibroblasts, hair bulbs, or bone marrow derived MSCs) (1, 6, 8, 12, 19, 27). When DNA variants are identified within germline range from DNA derived from these tissues, the result can be immediately interpreted as germline and is immediately relevant when considering relatives as allogeneic HSC donors (6). Some retrospective studies have determined germline status through sharing of the allele in relatives (15) or the presence of an allele at a VAF consistent with germline status obtained from hematopoietic tissue in clinical remission (28), but we do not advocate such approaches in general for clinical germline predisposition testing due to the concerns of somatic reversion and somatic mosaicism, as discussed above. When germline genetic testing is incorporated into the initial assessment of patients with HMs, results are often available when that individual and their family members are being evaluated for allogeneic HSCT and for optimal post-transplant prophylaxis (29).

The assay designs used in tumor-based molecular profiling versus germline testing are also quite distinct. Acquired mutations in HMs typically occur in gene exons, and therefore, platform designs tend to be capture-based amplification assays with hundreds-thousands fold depth to allow detection of small clone sizes (30). Also, these assays are typically designed to detect single nucleotide variants (SNVs), but can sometimes detect large copy number variants (CNVs). In contrast, germline variants exist as SNVs in exons and regulatory regions like promoters and enhancers, the latter being poorly covered generally in exon-based panels, as well as CNVs of various sizes. Tumor-based panels generally are not designed to detect small CNVs, and therefore, are incapable of detecting them.

In contrast, germline genetic testing platforms are designed to cover the mutation types and genomic elements where those variants occur (8), often performed as augmented whole exome sequencing (aWES) in which primers designed to capture non-coding regions are added to an exome platform, and bioinformatic pipelines capable of identifying CNVs from such data are used. Alternatively, germline platforms may combine aWES with microarray analysis or multiplex ligation amplification (MLPA) to detect CNVs (6). Some advocate performing whole genome sequencing (WGS) initially, which facilitates CNV detection, but the current cost of running and storing such data are prohibitive for most clinical centers (6, 31). Importantly, some platforms separate SNV from CNV testing, so careful attention must be paid at the time of test ordering to ensure that testing is comprehensive for both SNVs and CNVs. Generally, a sequencing depth of about 30-50X is sufficient to detect germline genetic variants. Finally, germline cancer predisposition genes continue to be discovered, especially for hematopoietic malignancies, and therefore, clinical testing platforms need to be flexible to accommodate the increasing number of genes recognized to confer risk.

## 7 Conclusion

The increasing use of molecular profiling of tumor cells for prognostication and therapy decisions affords the opportunity to identify DNA variants that are germline in nature and confer risk to hematopoietic, and potentially other, cancers. These panel-based tests do not substitute for proper germline genetic testing, which relies on platform designs that accommodate a growing list of cancer predisposition genes and a myriad of mutation types and rely on DNA that is equivalent to germline. Therefore, when a deleterious DNA variant is identified at an allele frequency consistent with the germline, especially when it is observed consistently across time and during remission, it should be considered as potentially germline in nature. Once prioritized for germline testing, individuals can undergo assessment in time for future treatments, such as allogeneic HSCT, which often involves relatives as the donor stem cell source.

## Author contributions

LG wrote this article and designed the tables and figures and is accountable for its contents of the work.
